# Editorial: Reactive oxygen species in chloroplasts and chloroplast antioxidants under abiotic stress

**DOI:** 10.3389/fpls.2023.1208247

**Published:** 2023-05-26

**Authors:** Michael Moustakas, Ilektra Sperdouli, Ioannis-Dimosthenis S. Adamakis

**Affiliations:** ^1^ Department of Botany, School of Biology, Aristotle University of Thessaloniki, Thessaloniki, Greece; ^2^ Institute of Plant Breeding and Genetic Resources, Hellenic Agricultural Organisation–Demeter (ELGO-Demeter), Thessaloniki, Greece; ^3^ Section of Botany, Department of Biology, National and Kapodistrian University of Athens, Athens, Greece

**Keywords:** oxidative stress, redox homeostasis, acclimation, chloroplast electron chain, cell death, signal interaction, transcriptome, photooxidative stress

An unavoidable consequence of aerobic metabolism by living cells is the production of reactive oxygen species (ROS) as regular cellular metabolic by-products. Plant cellular metabolism is continuously producing ROS, such as superoxide anion radical ( ${\text{O}}_{2}^{•-}$ ), hydrogen peroxide (H_2_O_2_), and singlet oxygen (^1^O_2_), at basal levels, which are unable to cause damage, as they are scavenged by different antioxidant mechanisms (Asada, 1999; Apel and Hirt, 2004; Moustaka and Moustakas, 2014). Biotic and abiotic stresses, such as metal toxicity, salinity, drought, chilling, UV-B radiation, and insects, result in an enhancement of ROS (^1^O_2_, ${\text{O}}_{2}^{•-}$ , H_2_O_2_, OH**^•^**) creation in plants due to disturbance of cellular homeostasis, which can result in oxidative stress (Moustakas, 2021; Sperdouli et al., 2021; Moustakas, 2022). Oxidative stress results from the imbalance between the production of ROS and the scavenging of their reactive intermediates by antioxidants (enzymatic and non-enzymatic), causing cellular damage that can lead to cell death (Mittler et al., 2004; Gill and Tuteja, 2010; Foyer, 2018). Thus, response of plants to this imbalance before the damage of their cellular structures is critical for maintaining high rates of photosynthesis and also for their survival (Moustaka et al., 2015; Foyer, 2018).

Chloroplasts are considered as one of the most important producers of ROS in plant cells and, more specifically, the light reactions of photosynthesis. Under most abiotic stresses, the absorbed light energy exceeds the energy that it can handle, and thus it can damage the photosynthetic apparatus. If this excess excitation energy is not quenched by the photoprotective mechanism of non-photochemical quenching (NPQ), increased production of ROS occurs, which can lead to oxidative stress (Müller et al., 2001; Foyer and Shigeoka, 2011; Moustakas, 2022). Abiotic stress-induced ROS accumulation is scavenged by enzymatic antioxidants, such as superoxide dismutase (SOD), ascorbate peroxidase (APX), monodehydroascorbate reductase, (MDHAR), dehydroascorbate reductase (DHAR), glutathione reductase (GR), glutathione peroxidase, (GPX), guaicol peroxidase (GOPX), glutathione-S- transferase (GST), and catalase (CAT), and non-enzymatic metabolites, such as ascorbic acid, glutathione, a-tocopherol, carotenoids, phenolic compounds, and flavonoids (Noctor and Foyer, 1998; Gill and Tuteja, 2010; Moustakas et al., 2022).

Despite their destructive activity, ROS are well-described as second messengers in a variety of developmental and cellular processes including resilience to abiotic stresses (Gill and Tuteja, 2010; Mittler, 2017; Noctor et al., 2018). The role of chloroplast antioxidants, which often have overlying or interrelated functions, is not to totally eliminate ${\text{O}}_{2}^{•-}$ , H_2_O_2_, and ^1^O_2_ but rather to achieve an appropriate balance between production and subtraction to match with the operation of photosynthesis and permit an efficient spread of signals to the nucleus (Foyer, 2018; Adamakis et al.). Singlet oxygen and H_2_O_2_ give rise to independent footprints, which usually are not antagonistic (Foyer and Noctor, 2013). These chloroplast-derived oxidative signals can activate regulatory networks to facilitate plants to sense and respond to biotic and abiotic stress conditions (Mittler et al., 2004; Gill and Tuteja, 2010; Foyer and Noctor, 2013; Mittler, 2017). ROS not only activate the plant’s defense mechanisms in order to cope with the oxidative stress but also are essential for redox sensing, signaling, and regulation of plethora physiological functions tightly accomplishing plant function and development (Mittler, 2017; Noctor et al., 2018; Adamakis et al.; Moustakas, 2022). It is now well recognized that maintaining a basal level of ROS in cells is essential for life (Mittler, 2017; Moustakas, 2022).

In this editorial article, we summarize the articles in this Special Issue, which will update readers on the subject and could be useful for scientists working on this Research Topic. Recent advances in the subject have been attractively presented. Anthocyanins, whose synthesis is enhanced by biotic and abiotic stresses, function as sunscreens by modifying the quantity and quality of captured light and for protecting from UV-B, for the defense against herbivores, as attracters of pollinators, for the protection from photoinhibition, for ROS scavenging, and as a stress signaling molecule (Gould et al., 2000; Moustaka et al., 2020). Kitao et al. reported that outer-canopy leaves protect themselves against photooxidative stress *via* anthocyanins, while simultaneously shading inner canopy leaves and protecting them from strong light and oxidative stress by shading (holocanopy hypothesis), contributing to efficient N resorption as a whole canopy.

By using RNA-sequencing, Moreau et al. studied gene transcription in tomato leaves treated with the chitooligosaccharides–oligogalacturonides (COS-OGA) elicitor FytoSave^®^, which induced plants to fend off biotrophic pathogens, and observed an upregulation of sequences that code for chloroplast proteins of the electron transport chain, especially photosystem I (PSI) and ferredoxin. They concluded (Moreau et al.) that plant defense induction by COS-OGA induces a long-term acclimation mechanism and increases the chloroplast electron transport chain to supply electrons that are needed to mount defenses against biotrophic pathogens, targeted to the apoplast, without compromising biomass accumulation.

In acidic soils with water-logged regions that are often affected by ferrous iron (Fe^2+^) toxicity, ROS production is crucial, causing the major yield-limiting factor of rice production. Regon et al. reported that under severe Fe^2+^ toxicity, the biosynthesis of amino acids, RNA degradation, and glutathione metabolism were induced, whereas phenylpropanoid biosynthesis, photosynthesis, and fatty acid elongation were inhibited, while ROS homeostasis was proposed as an essential defense mechanism under such conditions.

Although a great amount of information has been gathered over resent years about ROS production in plant cells, our understanding of how plants perceive ROS presence when triggered by different type external stress factors and how plants set priorities between different signals is still low (Mansoor et al., 2022). In an effort to elaborate ROS signaling, Xu et al. applied ozone (O_3_) and high light (HL) combinational treatments in *Arabidopsis thaliana* and analyzed gene expression and transcript profiles to distinguish the signaling effects that are orchestrated due to apoplastic and chloroplastic ROS increase. RNA-seq experiments identified three marker genes (*ELIP2*, *APX2*, and *ZAT12*) that displayed differential fold induction after HL, O_3_, or combined treatments, and further analysis showed that the regulation of their transcript levels responded differentially to signals that originated from inside the cell (chloroplast) and from outside (apoplast). Therefore, the study of Xu et al. further illustrated that signals from the different subcellular compartments have diverse signaling roles since O_3_ and HL caused differential expression of genes with contrasting roles. Moreover, the combined O_3_ + HL treatments emphasized that the apoplastic and chloroplastic ROS activated dissimilar signaling pathways, and one signal initiated from HL originated from the apoplastic ROS and regulated changes in transcript levels for genes related to pathogen infection and cell death.

Wang et al. studied changes in the transcript levels of a NAC transcription factor encoding the gene *LcNAC13* in Litchi trees when grown in low or high temperatures. ROS induced with methyl viologen further confirmed that *LcNAC13* transcription factor is involved in ROS-induced leaf senescence. It seems therefore plausible that *LcNAC13* transcription factor governs the expression of different genes and is a key regulator of leaf senescence.

The above studies clearly show the “genomic arsenal”, where plants have to sense and respond against diversely induced ROS signaling. Nevertheless, a lot remains to be discovered.

## Author contributions

All authors listed, have made substantial, direct, and intellectual contribution to the work, and approved it for publication.
